# CCL2 facilitates spinal synaptic transmission and pain via interaction with presynaptic CCR2 in spinal nociceptor terminals

**DOI:** 10.1186/s13041-020-00701-6

**Published:** 2020-11-23

**Authors:** Sui-Bin Ma, Hang Xian, Wen-Bin Wu, Shuo-Yao Ma, Yu-Ke Liu, Yu-Tong Liang, Huan Guo, Jun-Jun Kang, Ying-Ying Liu, Hui Zhang, Sheng-Xi Wu, Ceng Luo, Rou-Gang Xie

**Affiliations:** 1 Department of Neurobiology, Fourth Military Medical University, Xi’an, 710032 China; 2 Department of Orthopedics, Xijing Hospital, Fourth Military Medical University, Xi’an, 710032 China; 3 The Fourth Regiment, School of Basic Medicine, Fourth Military Medical University, Xi’an, 710032 China; 4 The Sixth Regiment, School of Basic Medicine, Fourth Military Medical University, Xi’an, 710032 China; 5 The Second Regiment, School of Basic Medicine, Fourth Military Medical University, Xi’an, 710032 China; 6 Pain and Related Diseases Research Laboratory, Medical College of Shantou University, Shantou, 515041 China; 7 Department of Health Statistics, Fourth Military Medical University, Xi’an, 710032 China

**Keywords:** Chemokines C, C motif chemokine ligand 2 (CCL2), C motif receptor 2 (CCR2), Spinal synaptic transmission, Nociceptor, Pain

## Abstract

Previous studies have shown that CCL2 may cause chronic pain, but the exact mechanism of central sensitization is unclear. In this article, we further explore the presynaptic role of CCL2. Behavioral experiments show that intervertebral foramen injection CCR2 antagonists into dorsal root ganglion (DRG) can inhibit the inflammatory pain caused by CCL2 in spinal cord. We raised the question of the role of presynaptic CCR2 in the spinal dorsal horn. Subsequent electron microscopy experiments showed that CCR2 was expressed in the presynaptic CGRP terminal in the spinal dorsal horn. CCL2 can enhance presynaptic calcium signal. Whole-cell patch-clamp recordings showed that CCL2 can enhance NMDAR-eEPSCs through presynaptic effects, and further application of glutamate sensor method proved that CCL2 can act on presynaptic CCR2 to increase the release of presynaptic glutamate. In conclusion, we suggest that CCL2 can directly act on the CCR2 on presynaptic terminals of sensory neurons in the spinal dorsal horn, leading to an increase in the release of presynaptic glutamate and participate in the formation of central sensitization.

## Introduction

There is growing evidence that neuroinflammation plays a key role in the pathogenesis of neuropathic and inflammatory pain. [1, 2]. Most known inflammatory mediators cause pain by binding to nociceptors, located in the peripheral nervous system [3–5]. Previous studies have demonstrated that chemokines are associated with chronic pain after nerve injury [6, 7] and chronic itch [8, 9]. Chemokines C–C motif chemokine ligand 2 (CCL2), also known as monocyte chemotactic protein 1 (MCP-1), which can recruit monocytes to reach inflammation, infection, trauma, ischemia and other sites. Chemokines C–C motif receptor 2 (CCR2) is the main receptor of CCL2. Evidence suggests that CCL2/CCR2 is involved in neuropathic pain [10, 11]. Zhang and De Koninck [12] found that CCL2 expression was increased in small- to large-diameter DRG neurons after ganglion ligation, which expressed nerve damage marker ATF-3. This result indicates that CCL2 expression was increased in injured neurons. Thacker et al. [13] found that after ligation of L5 nerve root, CCL2 can be produced in both the injured L5 and the undamaged L4 peripheral sensory neurons. Intrathecal injection of CCL2 causes inflammatory hyperalgesia [14]. DRG neurons can transport the generated CCL2 to the central terminal of the spinal cord through axoplasmic transport. CCL2 was found to be expressed in SP and CGRP-positive primary afferent fibers in the superficial dorsal horn of spinal cord [11, 15]. Nerve injury can induce CCL2 axoplasmic transport to the central terminal [13]. It is worth noting that the release of CCL2 in the spinal terminals of primary afferent neurons is activity-dependent. Strong electrical stimulation of dorsal root can quickly release CCL2 in the superficial layer of nerve-injured spinal dorsal horn [13]. However, the synaptic mechanism of CCL2-induced pain sensitization is not entirely clear.

Our previous study has shown that CCL2 can directly regulate the synaptic plasticity of excitatory neurons expressing CCR2 in the spinal cord lamina IIo, which is the basis for central sensitization in chronic pain [14]. We further demonstrate that CCL2 can directly interact with CCR2 to enhance NMDAR-induced currents, eventually leading to inflammatory pain mainly through CCL2-CCR2-pERK-GluN2B pathway [16]. However, there are still some issues that need to be further clarified. For instance, whether the presynaptic terminals express CCR2? What is the role of CCR2 in synaptic terminals? Utilizing patch clamp recordings together with biochemical as well as behavioral surveys, we demonstrated that CCL2 can directly act on the CCR2 located in presynaptic terminals of sensory neurons in the spinal dorsal horn, and leading to an increase in the release of presynaptic glutamate and participate in the formation of central sensitization.

## Methods

### Animals and pain model

C57Bl/6 background WT mice were purchased and bred in the Animal Facility of the Fourth Military Medical University. Young mice (4–6 weeks old, C57Bl/6) were used for electrophysiological studies in spinal cord slices. All the animal procedures were approved by the Animal Care Committee of the Fourth Military Medical university.

### Intrathecal administration

C57BL/6 mice were anaesthetized by 1% pentobarbital sodium. A midline incision was made along L2 to L4 vertebral plate and the muscle attached to spinous process removed [17]. With the tip of the sharp scissor, a 1-mm hole on the left vertebra was made until dura and clean CSF was exposed. An intrathecal catheter (polyethylene-10 tubing) was inserted from L3 and passed rostrally into the subarachnoid space until it reached L1/T13. After a flush with 10 μl saline, the exterior end of catheter was sealed by heat. Penicillin antibiotics were used to prevent infection at the end of intrathecal catheterization. The mice were allowed to recover for 3 days. Any mouse showing motor deficits would be excluded. CCL2(5 μl) diluted by normal saline was intrathecally applied 3 day after intrathecal catheter implantation.

### DRG injection of RS504393

C57Bl/6 mice were anaesthetized with 2% isoflurane. The procedure for intervertebral foramen injection of RS504393 was the same as described previously [18, 19]. In brief, the bilateral iliac spines were exposed to locate the L3 and L4 vertebrae of mice. The 26-gauge needle mated to a Hamilton syringe (Hamilton, Reno, NV) was inserted at a 45° angle at the intersection of the lower edge of the ipsilateral L3 and L4 vertebrae. There was a sense of restriction when the needle entered the transverse foramen, and the paw retraction reaction of mice was the sign of the needle entering the transverse foramen. The L3/L4 intervertebral foramen was hence infiltrated with RS504393 (1 μg/20 μg) or vehicle. RS504393 was suspended in a 1% DMSO/saline mix.

### Behavioral analysis

Animals were habituated in the testing environment for at least 2 d before baseline testing. Thermal hyperalgesia and mechanical allodynia were tested as previously described [14, 20]. The experimenters were blinded to treatments.

### Spinal cord slice preparation and patch-clamp recordings

As we previously reported [14], the Krebs’ solution contains (in mM): 127 NaCl, 3.6 KCl, 2.4 CaCl_2_, 1.3 MgCl_2_, 1.2 NaH_2_PO_4_, 26 NaHCO_3_, and 15 glucose. After establishing the whole-cell configuration, neurons were held at the potential of + 40 mV to record NMDAR evoked EPSCs (NMDAR-eEPSCs) by stimulating the dorsal root entry zone via a concentric bipolar electrode using an isolated current stimulator [20]. The internal solution contains (in mM): 110 Cs_2_SO_4_, 2 KCl, 0.1 CaCl_2_, 2 MgCl_2_, 1.1 EGTA, 10 HEPES, 5 ATP-Mg. QX-314 (5 mmol/L) was added to the pipette solution to prevent discharge of action potentials. Signals were filtered at 2 kHz and digitized at 5 kHz. Data were stored and analyzed with a personal computer using pCLAMP10 software (Molecular Devices).

### Immunofluorescence labelling

Immunohistochemistry was performed according to standard protocols, and the following primary antibodies were used: Isolectin B4 antibody (Biotinylated GRIFFONIA, 1:200, vector laboratories, B-1205), CGRP antibody (Goat, 1:300, Abcam, ab36001), NF200 antibody (mouse, 1:200, Sigma-Aldrich, N5389), CCR2 antibody (rabbit, 1:300, NOVOUS, NBP1-48,337), Alexa Fluor® 594 (donkey anti-rabbit IgG, 1:1000, Abcam, ab150132), Alexa Fluor® 488 (donkey anti-mouse IgG, 1:1000, Abcam, ab150105; donkey anti-goat IgG, 1:1000, Abcam, ab150129). Alexa Fluor 488-conjugated streptavidin to visualize the riboprobes (1:1000, Invitrogen, E13345).

### Drugs and administration

CCR2 antagonist, RS504393 (Tocris Bioscience, Bristol, UK); CCL2 (R & D Systems, Minneapolis, MN, USA) were used in this study.

### Injection of AAV virions in DRG in vivo

Virus injection in DRG was performed as described previously [21]. Briefly, mice were anesthetized with isoflurane and L3/L4 DRGs exposed by removal of the lateral processes of the vertebrae. The epineurium over the DRG was opened, and the glass pipette with fine tip was inserted into the ganglion, to a depth of 100–150 μm from the surface of the exposed ganglion. After waiting 2 min to allow sealing of the tissue around the pipette tip, 700 nl of virus solution was injected at a rate of 0.1 μl/min using microprocessor-controlled minipump (RWD). The pipette was removed after a further delay of 5 min. The muscles overlying the spinal cord was carefully sutured and mice allowed to recover at 37 °C warming blanket. Mice were allowed to recover for 4 weeks before commencing various tests.

### Calcium imaging with GCaMP6s in presynaptic terminals of nociceptors

As described above, rAAV-Ef1a-DIO-GCaMP6s-WPRE-pA was injected into L3/L4 DRGs of SNS-Cre mice, a mouse line expressing Cre recombinase under control of the Nav1.8 promoter. Four weeks after virus expression, transverse 350–450 μm- thick spinal cord slices with dorsal roots attached were obtained. GCaMP6s signal in the presynaptic terminal of nociceptors in the superficial spinal dorsal horn was visualized using an upright super-resolution Olympus FV1200 confocal microscope (Olympus, Japan). Images were acquired at 1 Hz. Fluorescence intensity of each puncta was measured. Bath application of CCL2 (100 ng/mL) was used to activate the Ca^2+^ signal in the presynaptic terminals of nociceptors.

### Fluorescence imaging with iGluSnFR in presynaptic terminals of nociceptors

rAAV-EF1a-DIO-iGluSnFR(A184S)-WPRE-hGH-pA was injected into L3/L4 DRGs of SNS-Cre mice as described above. 4 weeks after virus expression, transverse 350–450 μm thick spinal cord slices with dorsal roots attached were obtained. iGluSnFR signal in the presynaptic terminal of nociceptors in the superficial spinal dorsal horn was visualized using an upright super-resolution Olympus FV1200 confocal microscope (Olympus, Japan). Images were acquired at 1 Hz. Fluorescence intensity of each puncta was measured. Bath application of CCL2 (100 ng/mL) was used to activate the fluorescence signal intensity in the presynaptic terminals of nociceptors.

### Pre-embedding immunogold-silver cytochemistry

The protocol was adapted from the previous investigation [22]. Briefly, mice were deeply anesthetized and transcardially perfused with PBS followed by 4% paraformaldehyde and 0.05% glutaraldehyde in 0.1 M PB. The spinal cords were removed and postfixed in the same fixative for 4 h at 4 °C. Serial coronal sections at 100 μm thicknesses (VS1000s, Leica, Heidelberger, Germany). The 0.05% Triton X-100 was used for CCR2 immunogold-silver cytochemistry. After rinsing, the sections were postfixed in 2% glutaraldehyde in PBS for 45 min. Signals of CCR2 immunoreactivity were detected by silver enhancement kit in the dark (HQ Silver Kit, Nanoprobes). Sections were postfixed in 0.5% osmium tetroxide in 0.1 M PB for 2 h. They were dehydrated with graded ethanol, replaced with propylene oxide, and finally embedded in Epon 812 between plastic sheets. Flat-embedded sections were examined under the light microscope. The sections containing CCR2 immunoreactivity in spinal dorsal horn were cut with a diamond knife (Diatome, Hatfield, PA). After counterstaining with uranyl acetate, ultrathin sections were examined under the JEM-1230 electron microscope (JEOL LTD, Tokyo, Japan). Postsynaptic and presynaptic membrane, synaptic vesicles were measured using ImageJ (NIH) by observers blinded to the genotype of the samples.

### Statistical analysis

Differences between groups were compared using 1-way ANOVA or 2-way repeated measures ANOVA followed by Bonferroni’s test or by Student’s t test (2-tailed) if only 2 groups were applied. The criterion for statistical significance was *P* < 0.05.

## Results

### Ganglionic injection of RS504393 inhibits the mechanical and thermal hyperalgesia caused by intrathecal CCL2

**Fig. 1:**
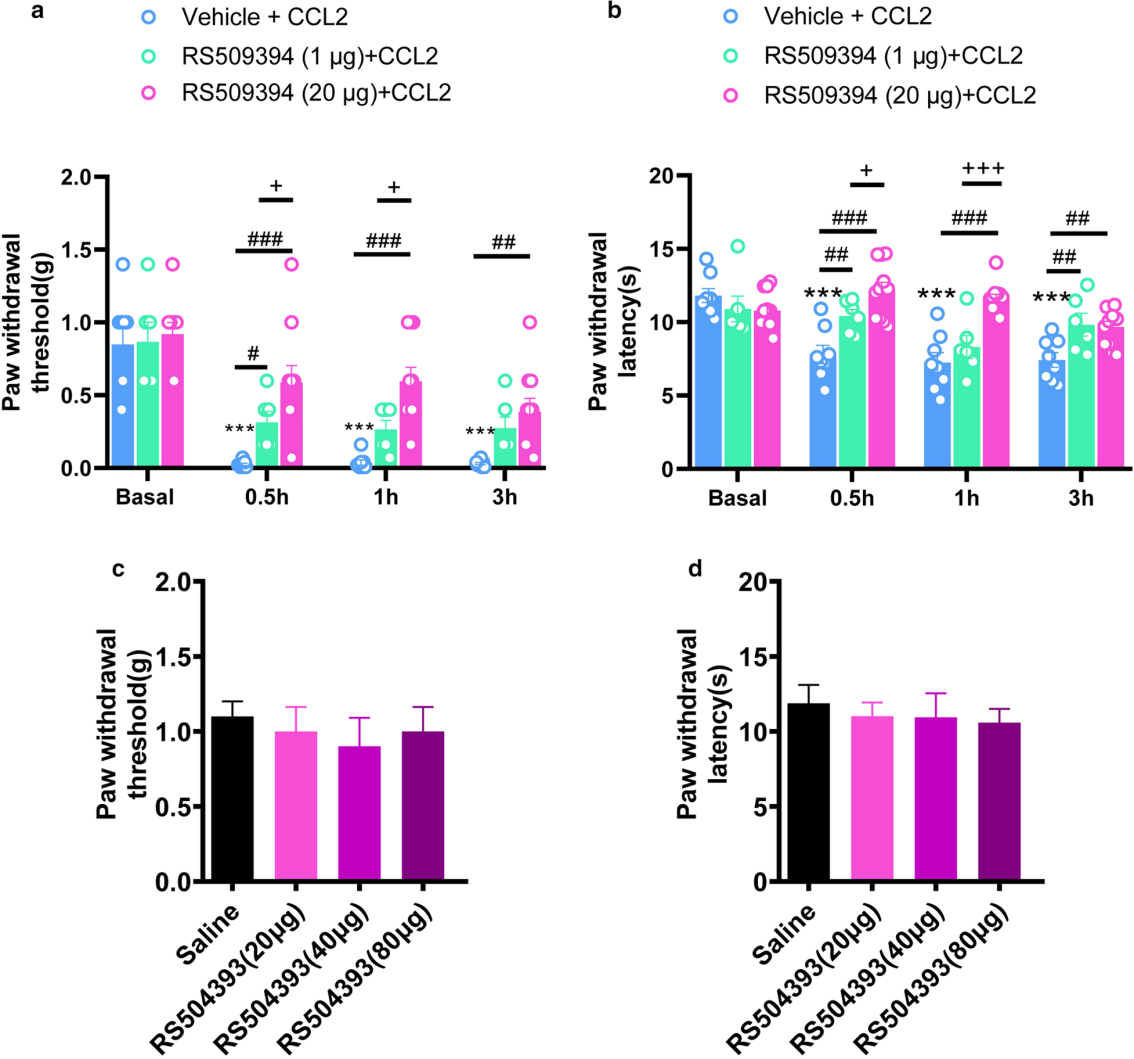
Ganglionic injection of RS504393 inhibits the mechanical and thermal hyperalgesia caused by intrathecal CCL2. **a** Prevention of CCL2 (100 ng, i.t.)-induced mechanical hyperalgesia by RS504393 (1 μg, 20 μg, intervertebral foramen injection (i.f.)). **b** Reversal of CCL2-induced heat hyperalgesia by RS504393 (1 μg, 20 μg, i.f.). **c**, **d** Basal mechanical threshold (**c**) and thermal latency (**d**) was not altered by RS504393 (20–80 μg, i.f). *and^#^and^+^*P* < 0.05, **and^##^*P* < 0.01, ***and^###^and^+++^*P* < 0.001. Two-Way ANOVA followed by post-hoc Bonferroni test (n = 10–11 mice/group). Data are expressed as mean ± SEM

Consistent with our previous study [14, 16], intrathecal CCL2 (100 ng) induced rapid mechanical hyperalgesia and thermal hyperalgesia, as characterized by a prominent drop in mechanical threshold and latency of paw withdrawal (Fig. 1 A&B, n = 10, *P* < 0.001). To further investigate the presynaptic mechanism of spinal cord central sensitization induced by CCL2, we apply DRG administration method to intervertebral foramen injection CCR2 blocker RS504393 (1 μg, 20 μg) into L3/L4 of DRG. Interestingly, ganglionic injection of CCR2 blocker RS504393 dose-dependently attenuated mechanical hyperalgesia and thermal hyperalgesia caused by intrathecal CCL2 (100 ng), respectively (Fig. 1a, b, n = 10, *P* < 0.05). In contrast, ganglionic delivery of RS504393 did not alter the basal mechanical threshold and thermal latency (Fig. 1c, d, n = 10, *P* > 0.05).

### CCR2 is widely expressed in DRG displays upregulation upon peripheral inflammation

**Fig. 2:**
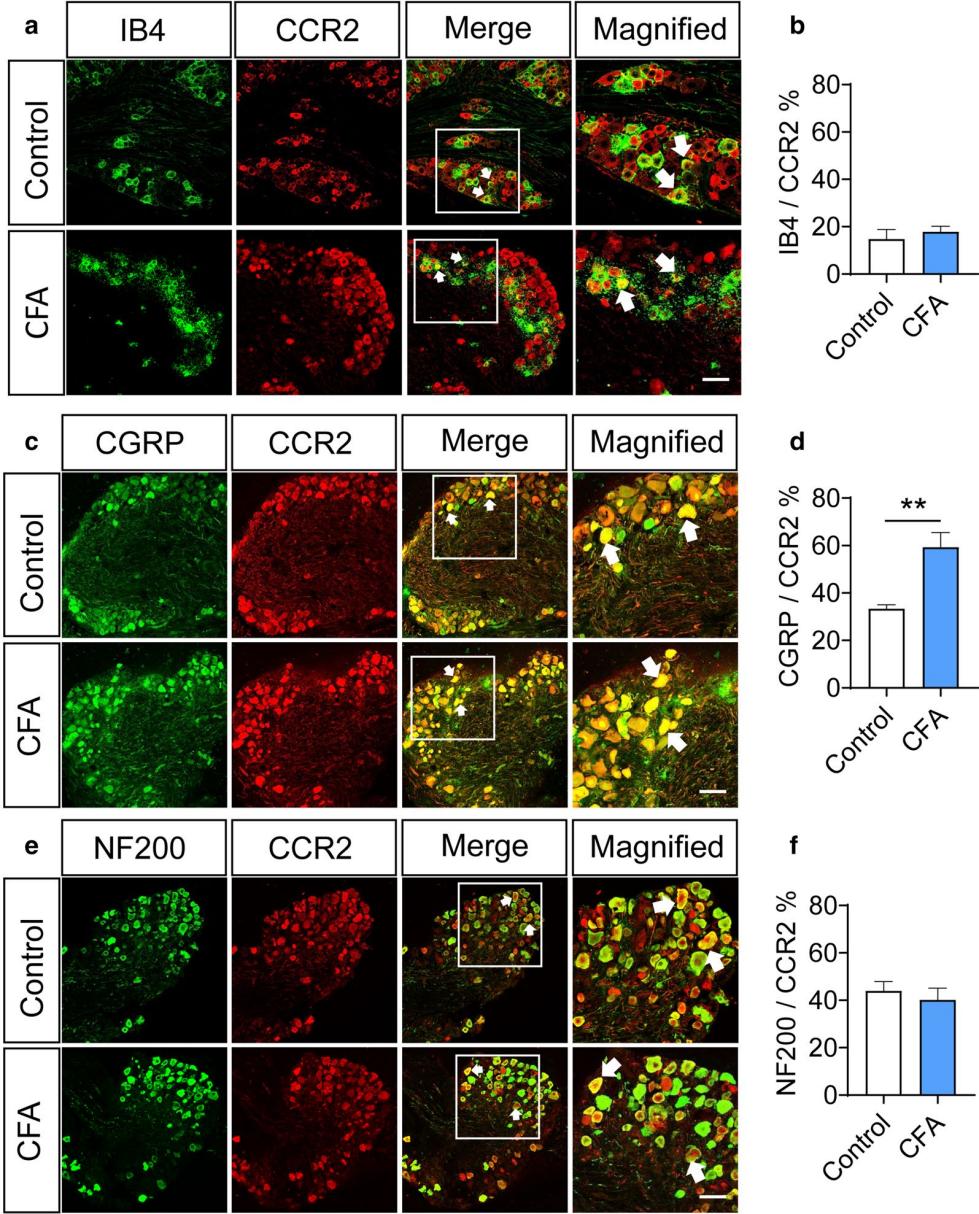
CCR2 is widely expressed in DRG neurons and displays upregulation following peripheral inflammation. **a**, **b** Typical examples and quantitative summary from dual immunofluorescence experiments showing CCR2 immunoreactivity IB4-binding non-peptidergic DRG neurons in the naïve and CFA-inflamed mice. **c**, **d** Immunofluorescence experiments and quantitative summary showing the coexistence of CCR2 immunoreactivity in CGRP-expressing peptidergic DRG neurons in naïve mice and its upregulation in CFA-inflamed mice. **e**, **f** Immunofluorescence staining and quantitative summary showing the colocalization of CCR2 immunoreactivity with NF200-positive large-diameter DRG neurons in naïve mice and no significant change in expression level after CFA inflammation. Note that in CFA-inflamed group, ipsilateral L3/L4 DRGs were obtained and analyzed at 24 h after intraplantar CFA injection. Arrows indicate the co-expressing cells. **P < 0.01. Two-Way ANOVA followed by post-hoc Bonferroni test (n = 4 mice/group). Data are expressed as mean ± SEM

In order to further verify that CCL2 can act on the presynaptic terminals in the superficial spinal dorsal horn, we employed immunohistochemical fluorescence methods to verify the expression of CCR2 in DRG. An anti-CCR2 antibody showed the wide immunoreactivity in DRG neurons of naïve mice. Confocal analysis revealed a colocalization of CCR2 in isolectin B4 (IB4)-labeled non-peptidergic nociceptors, CGRP-expressing peptidergic nociceptors as well as NF200-immunoreactive large-diameter sensory neurons (Fig. 2a, c, e). The co-staining rates of CCR2 with IB4, CGRP and NF200 are 14.7%, 33.3% and 44%, respectively in naïve state (Fig. 2b, d, f, n = 12 slices from 4 mice). Following peripheral inflammation induced by intraplantar CFA injection, the proportion of neurons co-labeled with CCR2 and CGRP was dramatically increased (Fig. 2c, d, 59.3% after CFA versus 33.3% control, n = 12, *P* < 0.01), while that of neurons co-labeled with CCR2 and IB4 or NF200 was not altered (Fig. 2a, b, e, f, *P* > 0.05, n = 12 slices from 4 mice).

### CCR2 is co-labeled with CGRP and IB4 in the superficial layer of spinal cord

**Fig. 3:**
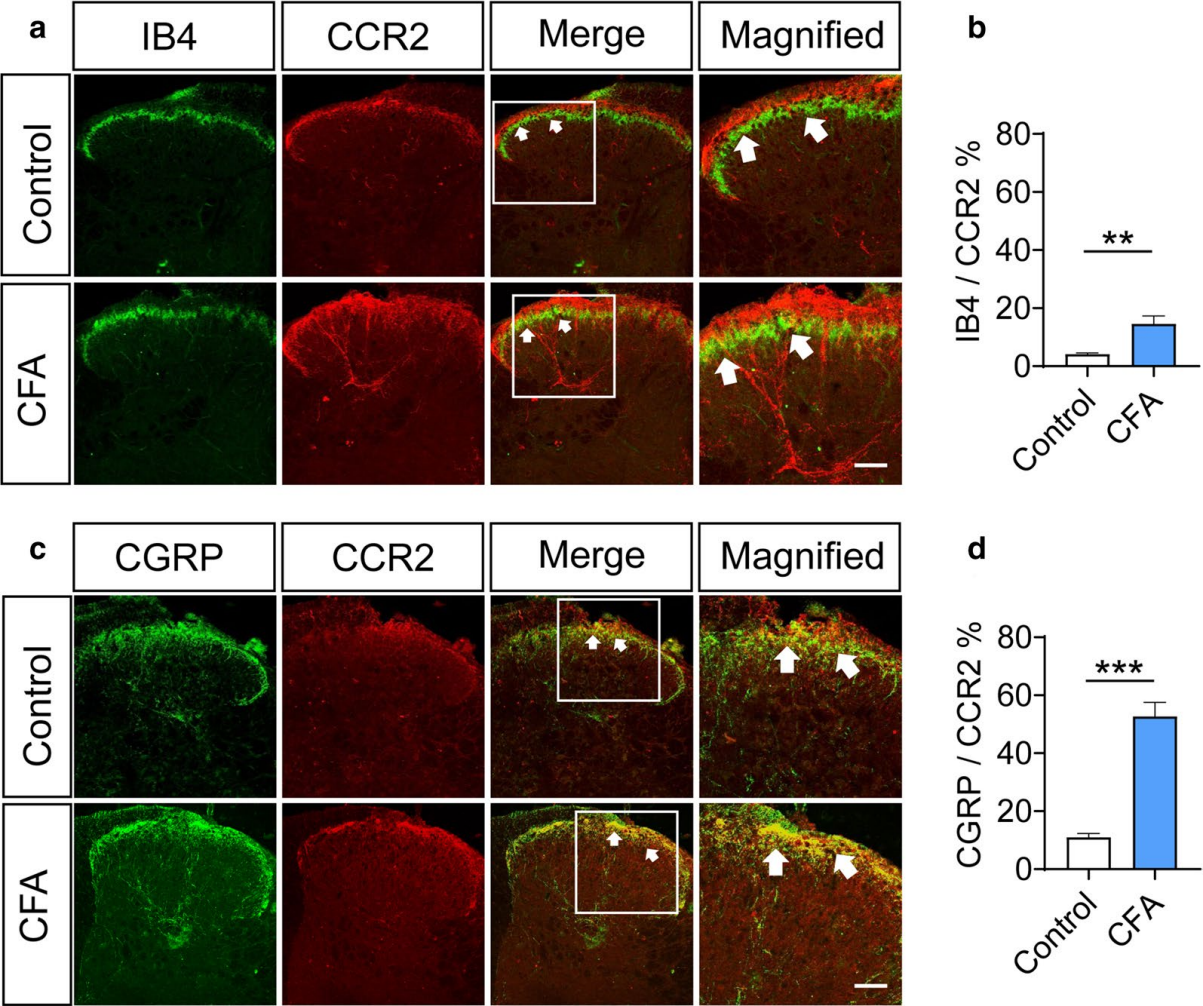
CCR2 is co-labeled with CGRP-expressing and IB4-binding terminals in superficial spinal dorsal horn and displays upregulation upon inflammation.** a**, **b** Immunofluorescence staining and quantitative summary showing an increase in CCR2-immunoreactivity in IB4-positive terminals in the ipsilateral (injured) dorsal horn at 24 h after CFA. **c**, **d** CCR2-immunoreactivity in CGRP-positive terminals are strongly increased in the ipsilateral (injured) dorsal horn at 24 h after CFA. Arrows indicate the co-expressing terminals. **P < 0.01, *** *P* < 0.001. Two-Way ANOVA followed by post-hoc Bonferroni test (n = 4 mice/group). Data are expressed as mean ± SEM

In order to further verify that CCL2 can act on the CCR2 receptor of the presynaptic terminal in the superficial spinal dorsal horn, we used the immunohistochemical fluorescence method to further verify the co-staining of the central terminal of DRG and CCR2. Our results showed that in the superficial layer of spinal cord of naïve mice, CCR2 is sparsely expressed in IB4-labeled non-peptidergic nociceptive terminals (4.1% of co-staining rate), but strongly with CGRP-expressing peptidergic nociceptive terminals (10.94% of co-staining rate) (Fig. 3a–d). Upon hindpaw CFA inflammation, CCR2 expression in superficial spinal dorsal horn displayed significant upregulation (Fig. 3a, c). CCR2 starts to be expressed in IB4-labeled terminals in superficial spinal dorsal horn of CFA-inflamed mice (Fig. 3a, b, n = 12 slices from 4 mice, *P* < 0.01). It is noteworthy that CCR2 is profoundly upregulated CGRP-expressing nociceptive terminals after CFA inflammation (Fig. 3c, d, n = 12 slices from 4 mice, *P* < 0.001). These results suggest that CCL2 may act on CCR2 expressed in the central process terminals of sensory neurons.

### Ultrastructural localization of CCR2 in the presynaptic terminals of spinal dorsal horn

**Fig. 4:**
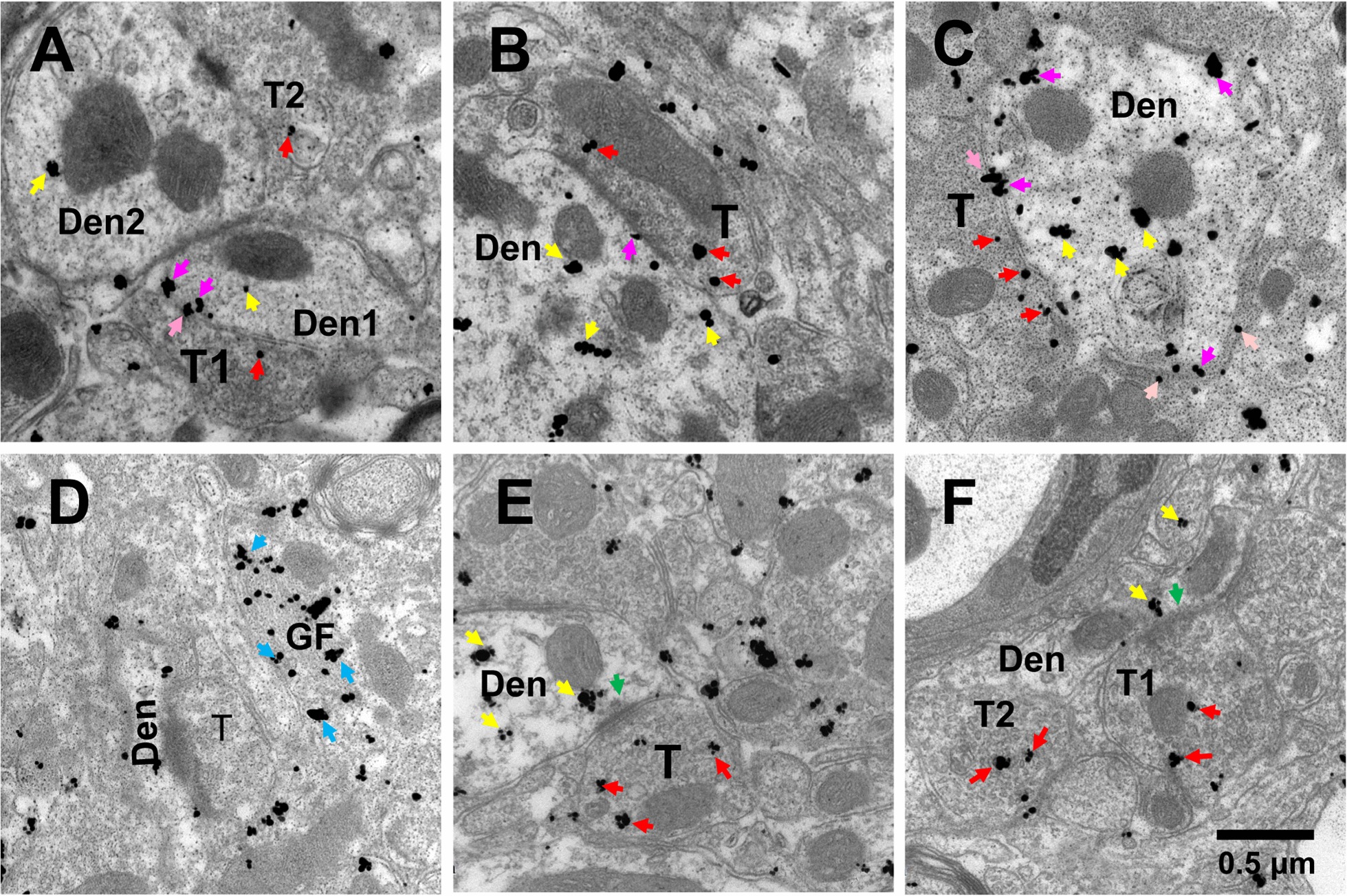
Ultrastructural preembedding double immunostaining showing the localization of CCR2 expression in the presynaptic terminal of the spinal cord. **a**–**c** Immunogold particles indicating that CCR2 immunoreactivity is mainly localized in the inside of the dendrites (yellow arrows), partly localized on the postsynaptic membrane (purple arrows), presynaptic membrane (pink arrows, **a**, **c**) and partly localized in presynaptic (red arrows). **d** Immunogold particles showing that CCR2 immunoreactivity is localized in glial filament (blue arrow). **e**, **f** Immunogold particles showing that CCR2 immunoreactivity is localized in the inside of the dendrites (yellow arrows) closing to synaptic vesicle (green arrows) and partly localized in presynaptic (red arrows). Den: dendrite, T: terminal, GF: glial filament. Scale bar = 0.5 μm

In order to prove that CCR2 exists in the central process of sensory neurons in the superficial spinal dorsal horn, we further applied the method of immunoelectron microscopy to label CCR2 with fluorescent gold in the spinal dorsal horn. The results showed that CCR2 not only exists in large numbers of postsynaptic neurons and glial cells in the superficial layer of the spinal dorsal horn, but also expressed on the presynaptic nerve terminals in the superficial layer of the spinal dorsal horn (Fig. 4a–f n = 4). At the same time, electron microscopy results showed that fluorescent gold-labeled CCR2 was expressed in presynaptic nerve endings with huge dense vesicles (Fig. 4a, e). Studies have shown that this kind of nerve endings are presynaptic nerve endings that express CGRP [23]. The above results further prove that CCR2 is expressed in CGRP-positive presynaptic nerve endings in the superficial spinal dorsal horn.

### CCL2 promotes presynaptic calcium signal enhancement via interaction with CCR2

**Fig. 5:**
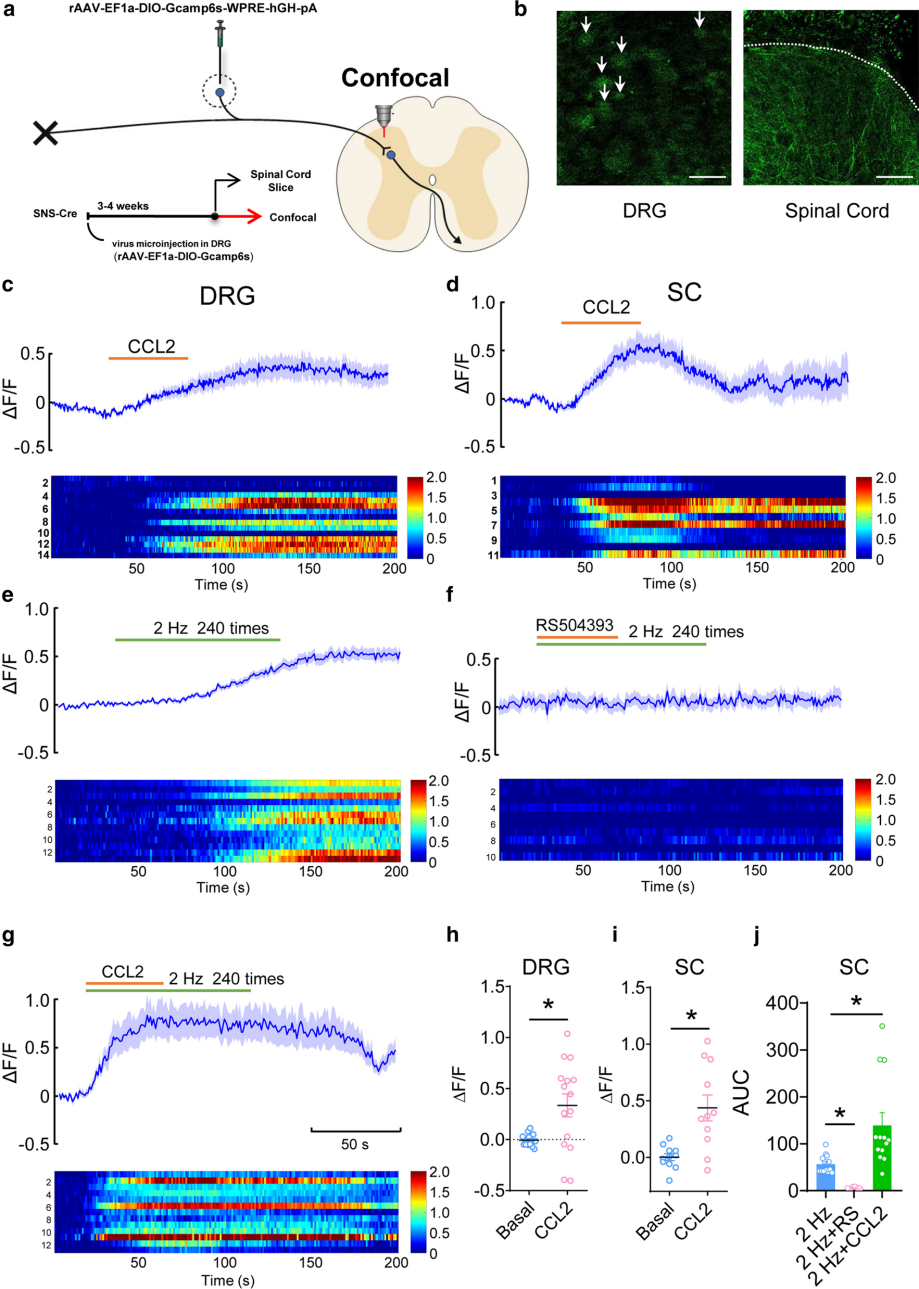
CCL2 promotes presynaptic calcium signals in spinal nociceptor terminals. **a** Schematic demonstration of the experimental approach for specific expression of GCaMP6s in L3/L4 DRGs in SNS-Cre mice and Ca^2+^ imaging in nociceptive DRG neurons and the presynaptic spinal terminals of nociceptors. **b** Photographs shown are typical examples of GCaMP6s expression in DRG neurons (Scale bar 50 μm) and the presynaptic nociceptor terminals in the spinal dorsal horn (Scale bar 10 μm). **c**, **h** Typical examples, heat map and quantitative summary showing that bath application of CCL2 directly induces the calcium signals in the nociceptive DRG neurons. **d**, **i** Representative traces, heat map as well as quantitative summary showing that perfusion of CCL2 directly induces the calcium transients in the presynaptic nociceptor terminals of superficial dorsal horn. **E**–**g** and **j** Typical images and heat map as well as quantitative summary showing the calcium signals induced by conditioning low frequency stimulation (**e**), with the presence of RS504393 (**f**) and CCL2 (**g**) in the presynaptic nociceptor terminals of spinal dorsal horn. *P < 0.05, two-Way ANOVA followed by post-hoc Bonferroni test (n = 10–14). Data are expressed as mean ± SEM

So, what function does CCR2 expressed in the superficial layer of the spinal dorsal horn have? It is assumed that abnormal increase of intracellular Ca^2+^ plays crucial roles in the development of chronic pain [24, 25]. We sought to ask whether CCR2 is involved in the mobilization of Ca^2+^ in DRG neurons and its presynaptic spinal terminals. To address this, we applied genetic technology and microinjection methods to inject the rAAV-EF1a-DIO-GCaMP6s-WPRE-hGH-pA virus into L3/L4 DRGs of SNS-Cre mice. 4 weeks later, we obtained the entire DRG and cut the spinal cord section attached with dorsal root at 350–400 μm-thickness. Confocal microscopy was used to observe the calcium signal changes in the DRG neurons and its presynaptic nociceptive terminals in superficial spinal dorsal horn (Fig. 5a, b). Our results showed that bath application of CCL2 can directly induce the calcium signals in the DRG (Fig. 5c, h, n = 14, *P* < 0.05) and the superficial presynaptic terminals of the spinal dorsal horn (Fig. 5d, i, n = 11, *P* < 0.05), as characterized by elevation of GCaMP6s fluorescence. This result suggests that CCL2-CCR2 can actively mobilize intracellular Ca^2+^ in nociceptive primary sensory neurons and presynaptic terminals.

Next, we went on to assess whether CCL2-CCR2 modulates the increase of Ca^2+^ induced by repetitive activation of nociceptors. Previous studies have shown that conditioning low frequency stimulation of dorsal root at C-fiber intensity produces synaptic LTP in spinal-PAG (periaqueductal gray) projection neurons, which is assumed to be a cellular basis for pain hypersensitivity [21, 26, 27]. As shown in Fig. 5e, conditioning stimulation of dorsal root (2 Hz, 3 mA, 2 min) elicited a prolonged Ca^2+^ enhancement in presynaptic nociceptive terminals, which was almost abolished by co-application of CCR2 inhibitor, RS504393 (50 nmol/L) (Fig. 5f, j, n = 10–13, *P* < 0.05). In contrast, cotreatment with CCL2 strongly facilitated the Ca^2+^ elevation induced by conditioning stimulation (Fig. 5g, j, n = 10–13, *P* < 0.05). Taken together, we can infer from the above that CCL2 promotes presynaptic Ca^2+^ signals via interaction with CCR2 localized in presynaptic spinal terminals of nociceptors.

### CCL2 facilitates presynaptic glutamate release by acting on presynaptic CCR2

**Fig. 6:**
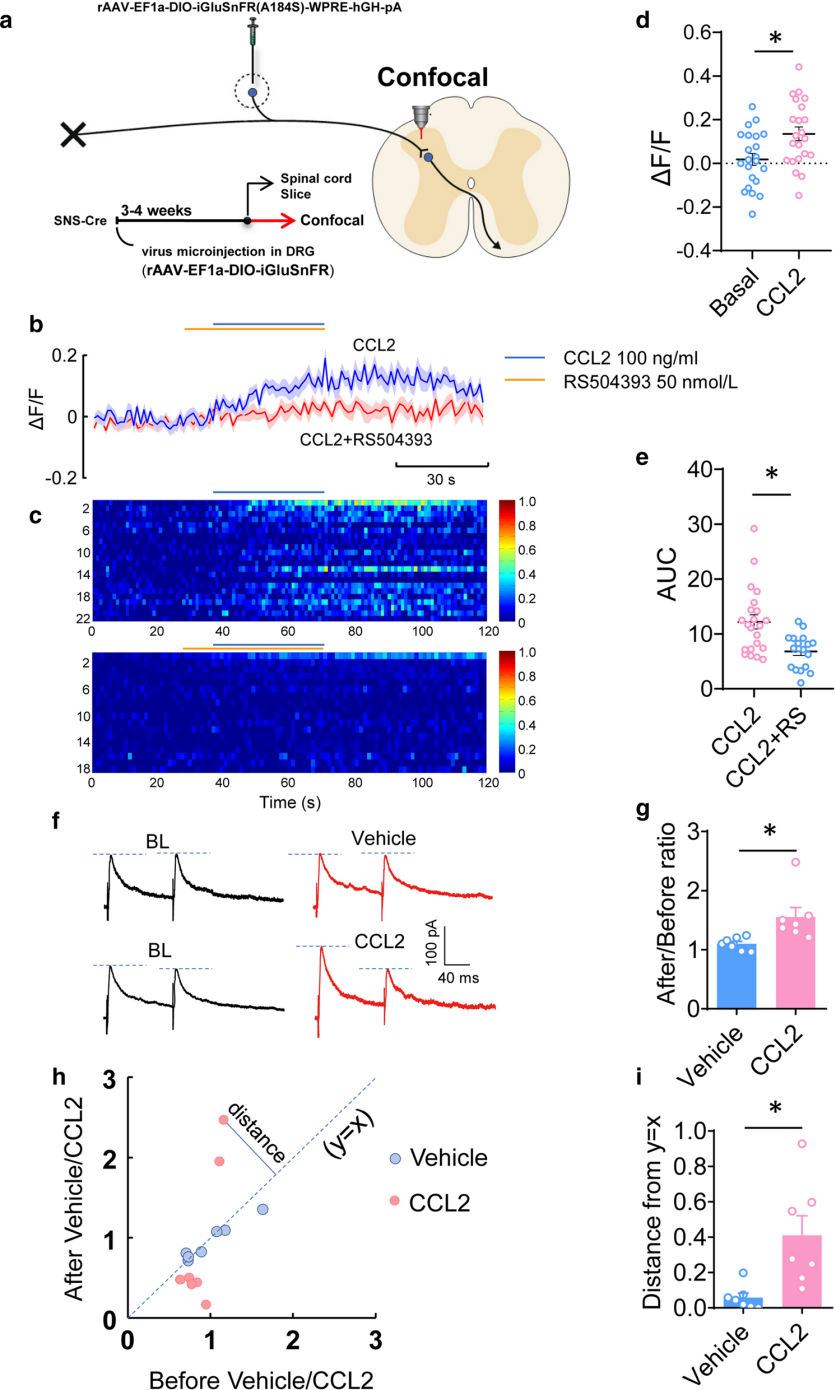
CCL2 facilitates presynaptic glutamate release by acting on presynaptic CCR2**. a** Schematic demonstration of the experimental approach for specific expression of GCaMP6s in L3/L4 DRGs in SNS-Cre mice and Ca^2+^ imaging in nociceptive DRG neurons and the presynaptic spinal terminals of nociceptors. Schematic diagram showing injection of rAAV-EF1a-DIO-iGluSnFR(A184S)- WPRE-hGH-PA into the DRG of SNS-Cre mice for monitoring presynaptic glutamate release from nociceptive primary sensory neurons. **b**, **c** Typical traces (**b**) and heat map (**c**) showing that bath application of CCL2 significantly enhances the fluorescence signal intensity in the superficial layer of the spinal dorsal horn, which is blocked by the presence of RS504393. **d** Quantitative summary of fluorescence change. **e** Quantitative analysis of area under curve (AUC). **f**, **g** CCL2 significantly increases the amplitude of NMDAR-eEPSCs. **h** Paired-pulse ratio (PPR) before and after application of CCL2/veicle are plotted in coordinate system. **i** Quantification analysis showing a clear change of PPR induced by CCL2 as compared to vehicle. *P < 0.05, two-way ANOVA followed by post-hoc Bonferroni test (n = 7–22). Data are expressed as mean ± SEM

Since transmitter release is known to be Ca^2+^ dependent, CCL2-induced Ca^2+^ influx and Ca^2+^ enhancement may lead to increased transmitter release and synaptic potentiation. To address this possibility, we further applied genetic methods to inject rAAV-EF1a-DIO-iGluSnFR(A184S)-WPRE-hGH-pA virus into L3/L4 DRGs of SNS-Cre mice for monitoring presynaptic glutamate release from nociceptive primary sensory neurons (Fig. 6a). As shown in Fig. 6b, bath application of CCL2 (100 ng/mL) elicited dramatic release of glutamate from presynaptic terminals, manifesting as a gradual increase of fluorescence (Fig. 6b–d, n = 18–22, *P* = 0.0077). Pretreatment with CCR2 inhibitor, RS504393 (50 nmol/L) produced a marked blockade of fluorescence enhancement, indicating that CCL2 facilitates presynaptic glutamate release via interaction with CCR2 (Fig. 6b, c, e n = 18–22, *P* = 0.0015).

Our previous studies have shown that CCL2 acts on the excitatory neurons of Lamina II in the dorsal horn of spinal cord, and produces hyperalgesia by enhancing the NMDA currents of Vglut2^+^ and SOM^+^ neurons [14]. In this process, does CCL2 act on pre-synapses and play a role? We used the whole-cell patch clamp experiment to detect the response of spinal cord Lamina II neurons to CCL2. We also performed the paired-pulse ratio (PPR) analysis, which represents a short-term plasticity and is well accepted as an indication of presynaptic mechanisms of long-term potentiation in the hippocampus [28]. Consistent with our previous report [14], bath application of CCL2 significantly increased the amplitude of NMDAR-mediated eEPSCs (Fig. 6f and g, n = 7, *P* = 0.0183). In addition to the postsynaptic mechanisms involved in this process [14], we are interested to know whether presynaptic mechanisms of CCL2 are involved as well. To this end, we plotted the paired-pulse ratio (PPR) of the entire cohort of recorded neurons before and after application of CCL2 (Fig. 6h). The spots falling in the diagonal line represent no significant change of PPR, the vertical distance of each spot to the diagonal line is manifested as the magnitude of change of PPR. Quantification analysis revealed that application of CCL2 showed much stronger changes of PPR, as compared to application of vehicle (Fig. 6i, n = 7, *P* = 0.0091). The above results suggest that CCL2 may act on presynaptic CCR2 and lead to an increased probability of glutamate release, which in turn results in synaptic potentiation and ultimately pain hypersensitivity.

## Discussion

Our study found that intervertebral foramen injection CCR2 antagonists into DRG can inhibit the inflammatory pain caused by CCL2. Subsequent electron microscopy experiments showed that CCR2 was expressed in the presynaptic CGRP terminal in the dorsal horn of the spinal cord. Furthermore, CCL2 can directly induce the calcium signals in the DRG and the superficial presynaptic terminal of the spinal dorsal horn, CCL2 can also enhance presynaptic calcium signal by 2 Hz stimulation. Whole-cell patch-clamp recordings showed that CCL2 can enhance NMDAR-eEPSCs through presynaptic effects, and further application of glutamate sensor method proved that CCL2 can act on presynaptic CCR2 to increase the release of presynaptic glutamate. In conclusion, we prove that CCL2 can directly act on the CCR2 on presynaptic terminals of sensory neurons in the dorsal horn of the spinal cord, lead to an increase in the release of presynaptic glutamate and participate in the formation of central sensitization.

Although CCL2 can recognize a variety of chemokine receptors, including CCR1, CCR2 and CCR4 [2, 29, 30], CCR2 is its main receptor [30, 31]. Similarly, CCR2 can also bind a variety of chemokines, including CCL2, CCL7 and so on. In mouse tissues, the ability of CCR2 to bind to CCL2 is ten times that of CCL7 [30]. Tanaka et al. [32] reported that after partial sciatic nerve ligation (PNSL), CCL2 on DRG neurons was rapidly upregulated (less than 4 h). In situ hybridization experiments showed that CCR2 mRNA was up-regulated in the compressed segment (L4/L5) and the adjacent non-compressed segment (L3/L6) after chronic compression of DRG [33]. CCR2 is up-regulated in the DRG expression of sciatic nerve demyelination model [31, 33, 34]. It fully shows that the up-regulation of CCR2 on DRG is an important factor in inducing pain. However, it is not clear whether the up-regulation of CCR2 on DRG cells can be transmitted to the terminal and play a role in the process of central sensitization. Our study further proves that CCR2 is highly expressed in the sensory neurons of the dorsal horn of the spinal cord after inflammatory injury, which may be an important reason for central sensitization caused by inflammation.

On isolated spinal cord slices, the application of CCL2 can rapidly increase the frequency of sEPSCs in spinal dorsal horn lamina II neurons [11], suggesting that CCL2 may enhance the release of glutamate through the presynaptic mechanism [8, 35, 36]. However, the reason why CCL2 causes the release of presynaptic glutamate is not clear. We have used electron microscopy, virology, glutamate sensor and other methods to further verify that CCL2 can act on the presynaptic CCR2 receptor, thereby enhancing the release of presynaptic glutamate.

It has become clear that neuroinflammation, mainly mediated by pro-inflammatory cytokines and chemokines, plays an important role in the establishment and maintenance of neuropathic pain. Previous studies have found that the chemokine CX3CL1 is expressed in primary afferent nerves and spinal cord neurons, and induces microglia activation through its microglia receptor CX3CR1 (neuron to microglia signaling) [37]. CCL2 are expressed in spinal cord astrocytes and act on CCR2 in spinal cord neurons to increase excitatory synaptic transmission (astrocytes to neurons signaling) [37]. Our research further found that CCR2 is expressed in the primary afferent nerve terminal, suggesting that the signal transduction of astrocytes to neurons also directly promotes the synaptic release of the primary afferent terminal of the spinal dorsal horn. There is also possibility that the effects were induced by interaction of CCL2 and CCR2 in postsynaptic neurons or glia cells indirectly.

Although studies have shown that CCL2 is highly expressed in IB4-positive nerve terminals in zymosan-induced hyperalgesia [38], studies have also shown CCL2 is mainly expressed on IB4-positive and CGRP-positive neurons in DRG [39]. Our research further clarified that CCR2 is mainly expressed in CGRP-positive DRG neurons and nerve terminals, and CCR2 expression is up-regulated after inflammatory pain model which further clarify the presynaptic target of CCL2 in the spinal cord. At the same time, we further explained the potential presynaptic analgesic mechanism of CCR2 as an analgesic target. A large number of studies have shown that DRG neurons are divided into different types according to their markers and excitability [40, 41]. Therefore, in which neuron subtype CCL2/CCR2 functions is worthy of further investigation.

Studies have shown that changes in presynaptic structure and function will lead to changes in synaptic plasticity in spinal cord [21]. Will the upregulation of presynaptic CCR2 cause changes in the synaptic plasticity of the spinal dorsal horn? This may be a potential mechanism of central sensitization.

CCR2 is also expressed in the pre-synapses of the superficial dorsal horn of the spinal cord. At the same time, under noxious injury conditions, the pre-synapses will also release CCL2. Meanwhile, the CCL2 released at the presynaptic terminal can directly act on the presynaptic CCR2, resulting in an increase in the probability of glutamate release, leading to central sensitization.

In summary, our present results demonstrate that CCR2 expressed in presynaptic nociceptor terminals is a key determinant for the initiation of central sensitization and further pain hypersensitivity associated with peripheral inflammation. Further mechanistic analysis reveals that injury or inflammation-induced production of CCL2 may directly interact with CCR2 in presynaptic nociceptor terminals, leading to increase of presynaptic glutamate release and participate in the formation of central sensitization, which in turn results in the exaggerated pain response. This study presents a strong basis for opening up a novel therapeutic target, CCR2 in nociceptors for treatment of pain hypersensitivity.

## Data Availability

The datasets used and/or analyzed in this study are available from the corresponding authors on reasonable request.

## Abbreviations

CCL2: Chemokines C–C motif chemokine ligand 2
CCR2: Chemokines C–C motif receptor 2
DRG: Dorsal root ganglion
eEPSCs: Evoked excitatory postsynaptic currents
MCP-1: Monocyte chemotactic protein 1
IB4: Isolectin B4
CFA: Complete Freund’s adjuvant
PAG: Periaqueductal gray
PPR: Paired-pulse ratio
NMDAR: NMDA receptor
